# A Glimpse into Genetic Diversity and Symbiont Interaction Patterns in Lichen Communities from Areas with Different Disturbance Histories in Białowieża Forest, Poland

**DOI:** 10.3390/microorganisms7090335

**Published:** 2019-09-09

**Authors:** Garima Singh, Martin Kukwa, Francesco Dal Grande, Anna Łubek, Jürgen Otte, Imke Schmitt

**Affiliations:** 1 Senckenberg Biodiversity and Climate Research Centre (SBiK-F), 60325 Frankfurt am Main, Germany; 2 Department of Plant Taxonomy and Nature Conservation, Faculty of Biology, University of Gdańsk, 80-308 Gdańsk, Poland; 3 Jan Kochanowski University in Kielce, Institute of Biology, 25-406 Kielce, Poland; 4 Department of Biological Sciences, Institute of Ecology, Evolution and Diversity, Goethe Universität, 60325 Frankfurt am Main, Germany

**Keywords:** barcoding, biological indicators, ITS, managed forests, species interaction network, photobiont

## Abstract

Anthropogenic disturbances can have strong impacts on lichen communities, as well as on individual species of lichenized fungi. Traditionally, lichen monitoring studies are based on the presence and abundance of fungal morphospecies. However, the photobionts, as well photobiont mycobiont interactions also contribute to the structure, composition, and resilience of lichen communities. Here we assess the genetic diversity and interaction patterns of algal and fungal partners in lichen communities along an anthropogenic disturbance gradient in Białowieża Forest (Poland). We sampled a total of 224 lichen thalli in a protected, a managed, and a disturbed area of the forest, and sequenced internal transcribed spacer (ITS) ribosomal DNA (rDNA) of both, fungal and algal partners. Sequence clustering using a 97% similarity threshold resulted in 46 fungal and 23 green algal operational taxonomic units (OTUs). Most of the recovered photobiont OTUs (14 out of 23) had no similar hit in the NCBI-BLAST search, suggesting that even in well studied regions, such as central Europe, a lot of photobiont diversity is yet undiscovered. If a mycobiont was present at more than one site, it was typically associated with the same photobiont OTU(s). Generalist species, i.e., taxa that associate with multiple symbiont partners, occurred in all three disturbance regimes, suggesting that such taxa have few limitations in colonizing or persisting in disturbed areas. *Trebouxia jamesii* associated with 53% of the fungal OTUs, and was generally the most common photobiont OTU in all areas, implying that lichens that associate with this symbiont are not limited by the availability of compatible photobionts in Central European forests, regardless of land use intensity.

## 1. Introduction

Lichens are frequently used as bioindicators for air pollution, climatic changes, and other anthropogenic disturbances [1,2]. The symbiotic association consisting of a fungal partner (mycobiont) and a photosynthetic partner (photobiont) is particularly sensitive to changes in substrate properties and microclimatic conditions. Anthropogenic activities in forests change host tree properties (e.g., stand age, and tree species composition), which has an effect, for instance, on the availability of bark with a particular texture or pH, as well as the microclimatic conditions, and can ultimately change the lichen community patterns of the regions under management [3,4]. Thus, lichens have been used as indicators of anthropogenic disturbance and forest health for decades [5,6,7].

Environmental assessments using lichens as indicators typically rely on presence and abundance data, based on morphological identification of the lichen-forming fungus [2,8,9,10]. However, morphological identification of specimens could bias diversity estimates, because cryptic (= molecular) diversity is not regarded. Furthermore, this approach neglects an assessment of the associated microalgae, which may also contribute to geographic distribution, and community composition of lichenized fungi [11,12]. For example, climatic factors can influence the altitudinal distribution of symbiotic green algae [13,14], or specific interaction patterns between mycobionts and photobionts [15,16]. Presently, we know much more about the diversity and distribution of fungal partners of lichens in forests [2,5,9,17,18]. However, a more comprehensive understanding of lichen communities requires an assessment of the diversities, distributions, and interactions of both partners since the availability of compatible algae is a prerequisite for the establishment and maintenance of lichen populations.

Molecular species delimitation has been effectively implemented in the last decades to uncover previously unknown lineages and to estimate species boundaries in both, lichenized fungi and their symbiotic algae [19,20,21,22,23,24]. In this regard, internal transcribed spacer (ITS) ribosomal DNA (rDNA) has been widely used as a barcode for both mycobionts [25,26,27], and their associated photobionts [24,26,28,29,30]. The availability of universal primers specific for the photobiont ITS have facilitated the identification of photobionts, often across different genera [31,32,33,34,35]. Nevertheless, photobionts of many lichens remain unidentified, hindering our understanding of patterns of symbiont interactions. Generating a molecular barcode of both partners in natural lichen communities will help us narrow down this knowledge gap and provide a more concise perspective on symbiont interaction patterns in lichens.

Symbiont interactions in lichens can be described in terms of specificity and selectivity. Specificity refers to the phylogenetic range of possible partners [36,37,38]. Selectivity refers to preferential association with a specific partner when more than one partner is available. Both specialist and generalist interactions have been reported for lichens [19,39,40,41]. It is expected that lichen communities harboring organisms with low selectivity and flexible partner choice may be more resilient to anthropogenic disturbances than communities consisting of specialized lichens. This is because loss of the photobiont species may not result in the immediate loss of the lichen due to flexible partner choice. The identification of both symbionts allows us to assess the interaction patterns present in a community. A better understanding of symbiont interaction patterns could help devise forestry practices that minimize the impact on lichen communities.

The objective of this study is to assess the impact of land use intensity on mycobiont and photobiont diversity and interaction patterns in lichen communities in a central European forest. For this, we generated ITS sequences of both symbiotic partners from lichens collected in a protected, a managed, and a disturbed area of Białowieża Forest, Poland.

## 2. Materials and Methods

### 2.1. Study Area and Sampling Design

The specimens for the present study were collected by AŁ and MK, in 2016 from trees located in three different areas in Białowieża Forest, which differ in the degree of land use intensity (Table S1). Białowieża Forest in Central-Eastern Europe is one of the largest remaining parts of an extensive temperate forest system that once constituted the natural vegetation of the European lowlands. The first study area (“protected”) is present in forest section No. 256 (52°45′45″ N 23°52′42″ E) and is the best-preserved forest ecosystem of Białowieża Forest comprising trees of different age, and dead wood present in the form of abundant logs and snags [10,42]. Some of the oldest trees in this area are 100 to 200 years old (oaks and hornbeam respectively). The second study area (“managed”) is located west of Budy village in forest section No. 335D (52°44′03″ N 23°42′59″ E) outside the national park. This area contains trees of different age, but no logs and snags, and only a few stumps of cut trees, indicating management activity. The oldest trees in this area are about 100 years old (*Picea abies*). The dominant trees are mostly young and mature spruce (*Picea abies*) and hornbeam trees (*Carpinus betulus*), a few mature oaks (*Quercus robur*) and aspen (*Populus tremula*), and very few lime trees (*Tilia cordata*). The third study area (“disturbed”) is located south of Czerlonka village in forest section No. 469C (52°41′16″ N 23°43′02″ E). This area is the most disturbed, located close to a road and buildings, without dead wood in the form of decaying logs or snags. Presence of several stumps with cut marks indicates past logging activities. The oldest trees are rare (pines and spruces, about 170 years old), and most of the trees in this area (e.g., hornbeam) are younger (about 100 years or less). A few apple trees, possibly dispersed from a neighboring orchard, are also present in this area.

Samples in the three areas were collected from about 60 living trees of different species from near the ground to about 2 m up the stem. From each tree 1 to 3 samples were collected. Detailed specimen information is given in Supplementary S1 (Supplementary Materials). Specimens are deposited in the following herbaria: The Jan Kochanowski University in Kielce (KTC) and the University of Gdańsk (UGDA).

### 2.2. DNA Sequencing and Alignments

Total genomic DNA was extracted from lichen thalli using the cetyl-trimethyl ammonium bromide (CTAB) method [43]. We amplified fungal and algal internal transcribed spacer (ITS) ribosomal DNA (rDNA) using general primers or taxon-specific primers [27,30,44,45] (Table S2). PCRs were carried out in a volume of 25 µL. Each reaction mix contained 2.5 µL buffer, 0.13 µL (0.65 U) TaKaRa ExTaq (Takara Bio Europe, SAS, Saint-Germain-en-Laye, France), 2.0 µL dNTP mix (2.5 mM each), 1.0 µL each (10 mM) of the primer set (forward and reverse), c. 20 ng of template, and 16 µL H_2_O. DNA concentration was measured with a nanophotometer (Implen) and by gel images. Reactions were performed under the following cycling conditions: Initial denaturation at 95 °C for 4 min, followed by 35 cycles of 95 °C for 30 s, 50 °C/60 °C for 30 s (annealing temperature for fungal ITS and *Trebouxia* ITS: 50 °C, for the *Trentepohlia* ITS: 60 °C) and 72 °C for 1 min, and final elongation at 72 °C for 5 min. PCR products were checked for amplification on 1% agarose gels and sequenced using the Big Dye Terminator v.3.1 Cycle Sequencing kit (Applied Biosystems, Foster City, CA, USA) as follows: 1 min at 95 °C, and 30 cycles of 10 s at 96 °C, 10 s at 50 °C, and 2 min at 60 °C. Products were purified and then detected on an ABI PRISM 3730 DNA Analyzer (Applied Biosystems). Sequences were assembled using GENEIOUS v.5.4 [46]. Gaps were treated as missing data and ambiguously aligned regions were excluded. The sequences are deposited in GenBank (accession numbers- fungal ITS: MN387000-MN387223; photobiont ITS: MN396904-MN397127).

Bands of expected size were extracted using the peqGOLD Gel Extraction Kit (PEQLAB Biotechnologie GmbH, Erlangen, Germany) and labeled for cycle sequencing using the Big Dye Terminator v.3.1 Cycle Sequencing kit (Applied Biosystems, Foster City, CA, USA) and sequenced as follows: 1 min at 96 °C, and 26 cycles of 20 s at 96 °C, 5 s at 50 °C, and 2 min at 60 °C. Products were purified using the Big Dye XTerminator Purification kit (Life Technologies, Foster City, CA, USA) and then detected on an ABI PRISM 3730 DNA Analyzer (Applied Biosystems). Sequences were assembled using GENEIOUS v.5.4 [46]. Gaps were treated as missing data and ambiguously aligned regions were excluded.

### 2.3. Species Identification, OTU Analysis, and Association Network

Fungal and algal OTUs were inferred using a 97% similarity threshold using vsearch v2.8.4, which uses a fast heuristic based on nucleotides shared by the sequences in order to identify similar sequences [47]. The delimited fungal OTUs were assigned a species name based on morphological identification, and NCBI BLAST hits of ≥97% similarity to sequences present in GenBank (Table S1). We implemented the following scheme for identifying fungal species: If the first NCBI BLAST hit was the same as morphological identification (based on >97% sequence similarity), the morphological identification was confirmed. If the ITS sequence of the identified species was not present in GenBank, and no significantly similar BLAST hits were retrieved, the morphological identification was retained. If the first BLAST hit corresponded to the morphological identification but the sequence similarity was <97%, the species was given the morphological name along with the suffix sp. 1 and so on.

Assigning a taxon name to the ITS sequence of a lichen photobiont using NCBI BLAST searches is impaired by the incompleteness of the data base, the lack of reliably identified algal species, and the presently unresolved phylogeny and systematics of the large and variable genus *Trebouxia*. Blast searches often result in several similar hits, and different putative species names for a sequence. To reliably allocate OTUs to an algal species we decided to use only the reference ITS sequences derived from algal cultures. We aligned our algal ITS dataset to the ITS sequences of the reference ITS sequences of *Trebouxia* and *Trentepohlia* from the (1) SAG culture collection, Göttingen, Germany (Sammlung von Algenkulturen der Universität Göttingen, University of Goettingen, Germany), which maintains the largest and most comprehensive culture collection of microalgae, consisting of about 1400 species belonging to 500 genera, representing almost all classes and phyla of eukaryotic algae and cyanobacteria, and (2) UTEX culture collection, Texas, USA (University of Texas, USA), which maintains cultures of about 3000 strains of algae. Overall, 40 ITS reference sequences of *Trebouxia*, and *Trentepohlia*, representing 26 species, were aligned with our algal ITS data set of 224 sequences representing 23 OTUs.

**Figure 1:**
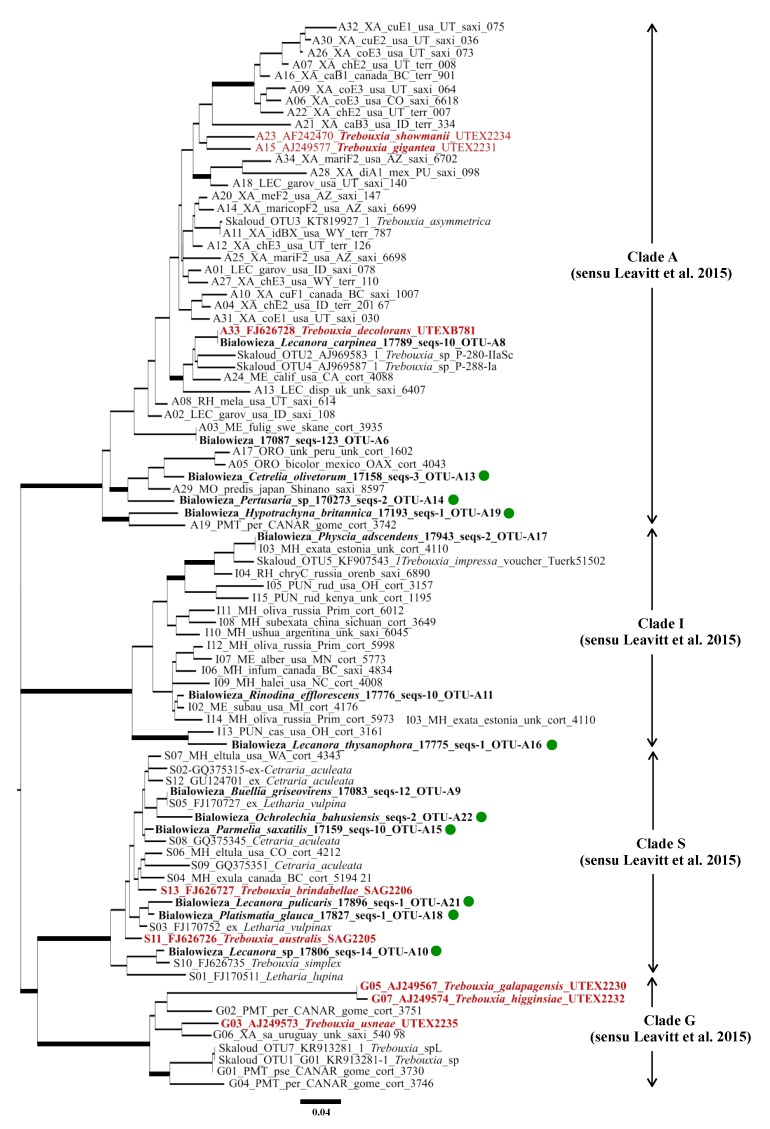
Internal transcribed spacer (ITS) gene tree constructed with the representative *Trebouxia* operational taxonomic units (OTUs) from this study, and 69 and seven representative OTUs from Leavitt et al. [19], and Škaloud et al. [48] respectively. Four major clades from Leavitt et al. [19] are indicated. ITS sequences from this study are in bold, and those from the SAG and UTEX culture collection are in red and bold. For the sake of reference to the already published dataset, the names of the representative OTUs from Leavitt et al. [19] and Škaloud et al. [48] are retained. The reference OTUs from this study are named according to the following scheme: Locality, DNA/sample ID, number of sequences of that OTU in the dataset, and lastly, *Trebouxia* OTU number. Novel OTUs are indicated with a green dot in front of the name of the representative OTU sequence.

In order to link the photobiont OTUs recovered in our study to the photobionts found in other studies, we performed a sequence-similarity based search in the NCBI database with 97% similarity as the threshold for the same photobiont OTU. This demonstrates, if the photobiont was reported from other lichens elsewhere. Additionally, to infer the other potential fungal hosts of the photobionts reported in our study, we generated a maximum likelihood tree with 1000 bootstraps from an alignment of the 14 representative *Trebouxia* OTUs from our study, eight representative *Trebouxia* OTUs from Škaloud et al. [48] and 69 representative *Trebouxia* OTUs from Leavitt et al. [19] (Figure 1).

The association network of the lichen-forming fungi and photobionts was inferred using the function plotweb v1.4.4 in the bipartite package in R [49,50].

## 3. Results

### 3.1. Genetic Diversity and Identification of the Lichen Symbionts

We detected 46 mycobiont and 23 photobiont OTUs in 224 lichen samples collected from the three areas in Białowieża Forest (Table 1). Between 25–32 fungal, and 11–17 algal OTUs were retrieved from each area in the Białowieża Forest (Table 1). We did not find high cryptic diversity in the fungal partners (Table S1 and Table 2). Only five fungal OTUs had no significant hit in GenBank, i.e., >97% sequence similarity (Table S1). In contrast, we found high cryptic diversity in the algal partners (Table S1 and Table 3). Only seven OTUs (out of 23) could be assigned a name based on clustering with the reference ITS sequences derived from the algal cultures deposited in SAG or the UTEX culture collection (Table 3), two algal OTUs had previously been reported from other lichens, but are not formally described, and 14 algal OTUs (10 *Trebouxia* and four *Trentepohlia*) had no significant hit in GenBank.

Out of 46 fungal OTUs, 11 belong to Parmeliaceae (nine genera), eight to Lecanoraceae (all to the genus *Lecanora*), four each to Pertusariaceae (all *Pertusaria*), Cladoniaceae (all *Cladonia*), Ramalineaceae (two each to the genera *Ramalina* and *Biatora*), and Graphidaceae (three *Graphis* and *Thelotrema lepadinum*), two each to Physciaceae (*Physcia adscendens* and *Rinodina efflorescens*) and Pyrenulaceae (both to the genus *Pyrenula*), and one each to Caliciaceae (*Buellia griseovirens*), Ochrolechiaceae (*Ochrolechia bahusiensis*), Ophioparmaceae (*Hypocenomyce scalaris*), Pilocarpaceae (*Fellhanera gyrophorica*), Phlyctidaceae (*Phlyctis argena*), Ropalosporaceae (*Ropalospora viridis*), and Sarrameanaceae (*Loxospora elatina*). As for the algal partners, out of 23 OTUs, 14 belonged to *Trebouxia*, five to *Trentepohlia*, and one each to *Apatococcus, Asterochloris*, *Dictyochloropsis,* and *Parachloroidium* (Table 3). *Trebouxia jamesii* was the most common photobiont associating with 16 mycobiont OTUs (out of 46) from six different families, followed by *Asterochloris phycobiontica* and *Trebouxia suecica*, each of which associated with seven mycobiont OTUs from three different families (Table 3).

The ITS phylogeny of the green algal genus *Trebouxia* generated from a combined dataset comprising representative OTUs from this study, Leavitt et al. [19] and Škaloud et al. [48] consisted of four supported clades sensu Leavitt et al. [19], namely Clade A (*T. arboricola*/*T. gigantea* clade), clade G (*T. galapagensis*/*T. usneae*), clade I (*T. impressa*/*T. gelatinosa*), and clade S (*T. simplex*/*T. letharii*/*T. jamesii*) Figure 1). From our study, six *Trebouxia* OTUs grouped within clade S, three within clade I, and five within clade A. None of the *Trebouxia* OTUs retrieved in our study clustered with Clade G (Figure 1).

### 3.2. Lichen Community Composition in the Three Regions

About 26% of the fungal and 30% of the algal OTUs respectively were shared among the three areas (Table 1). We found that 12 lichens were common to the three regions: *Buellia griseovirens*, *Lecanora argentata*, *L. carpinea*, *Lecidella elaeochroma*, *Melanelixia glabratula*, *Parmelia sulcata*, *Pertusaria amara*, *P. coccodes*, *P. leioplaca*, *Phlyctis argena*, *Platismatia glauca*, and *Ramalina farinacea* (Table 2). Lichens reported only from the protected area are *Pyrenula nitida*, and *Thelotrema lepadinum*. As for the photobionts, *Trebouxia jamesii* (OTU A6) was the most common photobiont in all the regions, overall associating with 53% of the sampled lichens (Table 3). Mycobiont species associating with *Trentepohlia* algae were more common in the protected area (Table 2).

### 3.3. Symbiont Interaction Patterns

**Figure 2:**
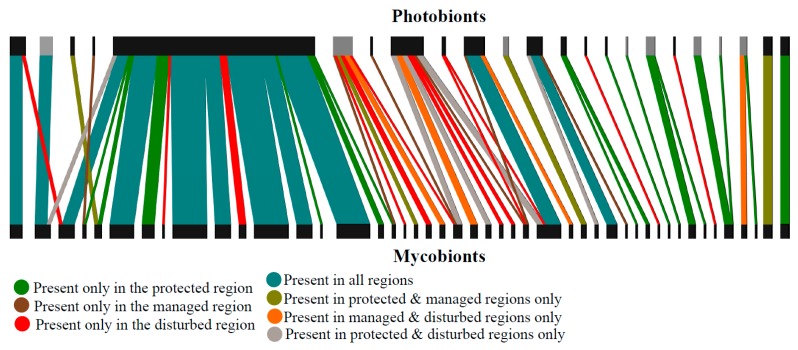
Association network based on fungal and algal ITS sequences, given a 97% similarity BLASTn threshold. Black and grey horizontal bars on the top represent *Trebouxia* and *Trentepohlia* OTUs respectively. Colored vertical bars represent fungal algal associations.

Several lichenized fungi and green algae associated with more than one partner, indicating the presence of many generalized lichen associations in Białowieża Forest (Figure 2). The bipartite network of symbiotic interactions between fungi and algae was asymmetric in species richness and included 46 fungal and 23 algal OTUs; resulting in a mean of two algae interacting per mycobiont (Figure 2). Among the mycobiont–photobiont associations, seven were one-to-one associations, and the rest involved more than two symbionts (Figure 2). Out of the seven one-to-one associations, three were unique to the protected region, two to the protected and managed region, and two were found only in the disturbed region. About 20% of the mycobionts (nine fungal OTUs) and 43% of the photobionts (10 photobiont OTUs) associated with more than one partner (Table 2 and Table 3).

Overall, we found 58 fungal–algal pairs, of which only 11 pairs were common to all regions (Figure 2, represented by blue colored vertical bars). Four fungal–algal pairs were present in the protected and managed regions and absent from the disturbed region (Figure 2, green colored vertical bars). The number of unique pairs was 16 in the protected, six in the managed, and 12 in the disturbed region.

## 4. Discussion

### 4.1. Genetic Diversity of Symbionts

Accurate recognition of fungal and algal lineages is important to understand the diversity patterns, specificity, and selectivity of symbionts [51]. In the case of the mycobionts, for most of the samples there were no conflicts between the morphological identification and the significant hits of the ITS sequence in GenBank database. For photobionts, only seven algal OTUs could be assigned to described species (Table 3). Fourteen photobiont OTUs had no significant similarity to ITS sequences in GenBank (Supplementary S1). Thus, our study highlights the presence a surprisingly large fraction of unknown diversity in the green algal genera *Trebouxia* and *Trentepohlia*, even in well studied ecosystems and geographic regions, such as forests in Central Europe.

Members of the genus *Trebouxia* constitute the most common lichenized algae associating with more than 20% of all lichenized fungi [52]. However, due to the paucity of taxonomically relevant morphological characters, only about 36 *Trebouxia* species have been formally described and deposited in the UTEX and SAG culture collections. Studies based on molecular data, however, indicate that the diversity of lichen-associated *Trebouxia* is high, with vastly undiscovered species-level diversity [19,30,53]. For instance, only 30% of the *Trebouxia* lineages which associated with 10 genera in Parmeliaceae could be allotted to described species [19]. Similarly, only 25% of the *Trebouxia* species which associated with the genus *Protoparmelia* (Parmeliaceae) could be assigned to previously described species [40]. Our study is in concordance with these studies suggesting the presence of vastly undiscovered species level diversity in *Trebouxia*.

### 4.2. Lichen Community Composition in the Three Regions

We found that 24% of the fungal and 30% of the algal OTUs were shared among all studied areas, suggesting that anthropogenic disturbance does not strongly affect these taxa (Table 2 and Table 3). Particularly those mycobionts associating with *Trebouxia jamesii* are not limited by the presence of a compatible photobiont in either disturbance regime, since this algal OTU was the most common photobiont in all studied areas. The protected area was characterized by the highest numbers of mycobiont and photobiont OTUs that were exclusively found in that area. One reason for this observation could be long-term ecological continuity and the presence of old trees in this habitat. Old trees provide variable microhabitat (e.g., bark with deep cracks; [7,54], stable substrate conditions over decades or centuries, i.e., longer periods of time available for colonization and growth [55], and stable microclimate, which can all lead to the development of a diverse lichen community. Some of the taxa we found in the protected area have been reported to preferentially occur on old trees (older than 180 years), such as *Lecanora glabrata*, *Pyrenula nitida*, and *Thelotrema lepadinum* and might be excellent indicators of long-term ecological continuity in forests [56]. Some of the taxa that are known to occur on younger trees (50–120 years old) we found in all three studied areas, i.e., *Buellia griseovirens*, *Lecidella elaeochroma*, *Parmelia sulcata*, and *Pertusaria amara* [56]. Overall, our study corroborates the importance of stand age for lichens [4,54,55,56].

Another reason for the higher number of unique fungal and algal OTUs in the protected region could be changes in the microclimatic conditions following anthropogenic disturbance. Epiphytic lichens are sensitive to microclimatic conditions, such as local humidity, and may change their vertical position on the trees [57,58]. As sampling was conducted only up to a maximum of 200 cm tree height in all sampling sites, taxa may have been missed that shifted higher up on the stem in response to anthropogenic activity.

The susceptibility of lichens to stand age-related properties renders the stand rotation length a crucial determinant of how severely forestry practices affect diversity loss. In fact, shorter rotation lengths have been associated with increased biodiversity loss of certain other organisms such as bryophytes [56,59]. Implementing the information on lichen substrate-age preference in decisions about rotation lengths, i.e., the duration between the plantation and final felling, may help mitigate biodiversity loss. Furthermore, apart from depleting the forest of old trees, many forestry practices lead to simplified forest structure with reduced diversity regarding trees species and age. Encouraging practices, which promote habitat diversity, for instance, retention practices, whereby living and dead trees are retained at final harvest, could be a way to minimize excessive habitat uniformity. Retention practices have also been shown to support higher biodiversity and a greater abundance of several species [60,61,62], including lichens [63,64,65]. Sustainable forestry practices encompassing various aspects of diversity and life-style traits of organisms could help avoiding future bottlenecks in habitat availability for lichens.

### 4.3. Symbiont Interaction Pattern

Our results indicate that generalist mycobionts and photobionts are present in all three areas of Białowieża forest. This could be attributed to the fact that generalist taxa sharing a common symbiont have a higher probability of finding a compatible partner and re-establishing after disturbance, provided the suitable microclimatic and substrate conditions are available. Identifying generalist taxa is simpler than identifying specialists, and often even low sampling might be sufficient to reveal low symbiont specificity. For instance, in our study we found that *Lecidella elaeochroma*, *Pertusaria leioplaca*, *Lecanora thysanophora*, *Loxospora elatina*, *Phlyctis argena, Platismatia glauca*, *Ochrolechia bahusiensis,* and *Thelotrema lepadinum*, associate with two or more than two photosynthetic partners (Table 2). The generalist interaction pattern of these mycobionts was revealed even if the number of samples per lichen was lower than 10. Furthermore, generalist taxa are more likely to be picked up under a random sampling scheme, because they are more common in the community. On the other hand, identifying highly specific taxa, i.e., one-to-one interactions, requires a larger sampling than that performed in the present study. A restricted sampling design may be misleading and may erroneously indicate a generalist as a specialist. For instance, although we found that *Lecanora argentata* (20 samples), *L. glabrata* (14 samples), *Melanelixia glabratula* (20 samples), and *Pertusaria amara* (19 samples) associate with only one photobiont species (Table 2), it is possible that they associate with a different photobiont species in more distant regions. The pattern observed in our study could be an artifact of photobiont-species availability. Samples from different geographic regions would be required to affirm if these lichens have high photobiont specificity and always associate with the same photobiont species as found in our study. Thus, identifying a highly specific mycobiont is more complex and is often obscured by sampling limitations. Interpretation on mycobiont specificity therefore must be ideally based on systematic, range wide sampling as the same lichen might be associated with a different photobiont species and vice-versa in different geographic regions.

Photobiont sharing is a common phenomenon in lichens [39,41,66,67]. Consequently, photobiont interaction patterns can also be inferred via by sequence-similarity based search to investigate if the same photobiont species was reported from other lichens. For instance, although *Trebouxia flava* (OTU A19) was recovered only once in our dataset (associated with *Physcia adscendens*; Table 3)*,* sequence-similarity search revealed that *Trebouxia flava* associates with *Physconia grisea*, *P. distorta*, *P. enteroxantha*, and *Tephromela atra* as well. This suggests that, in spite of its low occurrence in the Białowieża Forest, *Trebouxia flava* is a generalist, associating with multiple mycobionts across its range. Similarly, based on our dataset, we suggest that *Trebouxia* OTU A13 displays high specificity (although not exclusive one-to-one pattern) as it associates with only two mycobionts, *Cetrelia olivetorum* and *Menegazzia terebrata* (Table 3) and has not been reported to be associated with any other lichen so far.

Overall, mycobionts display high partner selectivity, whereas photobionts display low partner selectivity. For instance, *Trebouxia jamesii* associates with 16 different fungal OTUs, corresponding to 53% of the sampled lichens (Table 3). Most of the fungal partners of *T. jamesii,* however, associate only with *T. jamesii*. Lack of reciprocal high selectivity is a common phenomenon in lichens [19,38,40]. Low selectivity enhances the chances of the fungal partner to find compatible algae. For instance, *T. jamesii*-associating lichen-forming fungi might find it easier to retrieve the compatible partner from the photobiont pool and consequently their colonization in the disturbed region may not be limited by photobiont availability. This is particularly important for lichens, which have a phase in their reproductive cycle, during which fungi and algae disperse separately, e.g., in sexual reproduction after fungal spore release [31,68,69]. Thus, the processes of relichenization (fungal and algal partners joining to form a new lichen thallus) and recolonization of novel or disturbed habitats, might be comparatively easier for mycobionts associating with generalist algae. This might explain the presence of *T. jamesii*-associated lichens under all three disturbance regimes.

Out of 58 fungal-algal pairs, only 11 pairs were common to all regions (Figure 2, represented by blue colored vertical bars) whereas four others were present in the protected and managed regions only and absent from the disturbed region (Figure 2, green colored vertical bars). Each region harbored certain number of unique pairs, not present in the other regions. These findings are similar to those reported in other studies suggesting that distinct communities are present in the forest regions with different management types [64,70]. This could be attributed to the altered stand structure, habitat and microclimatic conditions, and/or differences in the photobiont pool among communities. Monitoring studies targeting these lichens are essential to infer the status of these fungal–algal pairs in the managed/disturbed regions.

Our study gives a preliminary account of the community structure of lichen mycobionts and photobionts in a temperate forest ecosystem. It provides baseline data on diversities and interactions of lichen symbionts, which can be informative for designing future studies involving lichen communities. More than half of the photobiont diversity presented here was sequenced for the first time, and many fungal–algal interaction pairs were shown for the first time. Initial assumptions that forest management practices could act as filters for particular photobionts, or that the same mycobiont associates with different algae in differentially disturbed areas could not be confirmed.

### 4.4. Impact of Forest Disturbances on Organisms and Communities

The magnitude of the impact of anthropogenic activities varies from organism to organism. This might depend on many factors including the life-cycle traits of a species. In this regard, a recent meta-analysis surveying lichen restoration after anthropogenic activities suggested that it might take up to 150–180 years for epiphytic lichen communities to reach a diversity similar to that of old-growth forests [67,71]. This is much longer than the average restoration time for other organisms, such as amphibians (<30 years; [72]), reptiles (<20 years; [72]), ectomycorrhizal fungi (90 years), epiphytic plants (100 years), and some common forest animals (average 20–40 years for bats, birds, and invertebrates; [73]). In general, restoring lichen communities might take longer because of multiple factors; (a) symbiotic lifestyle, (b) high partner specificity, (c) substrate or host tree specificity, (d) forest age (old forests harbor different microhabitats, light, and moisture conditions than managed/disturbed regions), and (e) slow growth rate.

## 5. Conclusions

Our study provided a glimpse into the lichen community composition, shared taxa among disturbance regimes and symbiont interaction patterns present in the different disturbance regimes of the Białowieża Forest. Furthermore, it provides an account of the photobiont partners of several lichens for the first time. This is valuable information when interpreting the symbiont interaction patterns of lichens from other communities. We suggest that the colonization and reestablishment of generalist taxa may be simpler after disturbance of due to higher chances of obtaining the compatible partner from the photobiont pool of the community.

## Figures and Tables

**Table 1:** OTUs recovered from the forest lichen community.

Site	# Samples	# Fungal OTUs	# Algal OTUs	Fungal OTUs Shared	Algal OTUs Shared	% Unique F OTUs	% Unique A OTUs
protected	78	32	17 (6)	NA	NA	34.3%	35.3%
managed	75	25	13 (2)	NA	NA	4%	15.4%
disturbed	71	27	11	NA	NA	27%	27.3%
overall	224	46	23	12	7	NA	NA

**Table 2:** Fungal–algal associations of lichens in a protected, a managed, and a disturbed area of Białowieża Forest.

Fungal OTU	Overall Frequency Fungal OTU	Protected		Managed		Disturbed	
		Frequency Fungal OTU	Associated Algal OTU(s)	Frequency of Fungal OTU	Associated Algal OTU(s)	Frequency Fungal OTU	Associated Algal OTU(s)
*Biatora mendax*	1	1	*T. jamesii*	0	--	0	--
*Biatora* sp. 1	2	2	*Apatococcus* sp.	0	--	0	--
*Buellia griseovirens*	14	2	*T. suecica, T. simplex*	4	*T. simplex*	8	*T. suecica, T. simplex, Trebouxia* (A22)
*Cetrelia olivetorum*	2	2	*Trebouxia* (A13)	0	--	0	--
*Cladonia chlorophaea*	1	0	--	0	--	1	*Asterochloris phycobiontica*
*Cladonia coniocraea*	3	0	--	2	*Asterochloris phycobiontica*	1	*Asterochloris phycobiontica*
*Cladonia digitata*	2	1	*Asterochloris phycobiontica*	1	*Asterochloris phycobiontica*	0	--
*Cladonia fimbriata*	3	0	--	0	--	3	*Asterochloris phycobiontica*
*Evernia prunastri*	4	0	--	0	--	4	*T. jamesii*
*Fellhanera gyrophorica*	5	2	*Parachloroidium* sp.	3	*Parachloroidium* sp.	0	--
*Graphis betulina*	1	1	*Trentepohlia* (A4)	0	--	0	--
*Graphis* sp. 1	3	2	*Trebouxia* (A11)	1	*Trentepohlia* sp SAG11880 (A1)	0	--
*Graphis* sp. 2	3	0	--	2	*Trentepohlia* (A4)	1	*Trentepohlia* (A4)
*Hypocenomyce scalaris*	2	0	--	0	--	2	*T. suecica*
*Hypogymnia physodes*	4	0	--	1	*T. suecica*	3	*T. suecica*
*Hypotrachyna revoluta*	1	1	*Trebouxia* (A18)	0	--	0	--
*Lecanora argentata*	20	4	*T. jamesii*	12	*T. jamesii*	4	*T. jamesii*
*Lecanora carpinea*	7	1	*T. decolorans*	3	*T. decolorans*	3	*T. decolorans*
*Lecanora chlarotera*	1	0	--	0	--	1	*T. jamesii*
*Lecanora glabrata*	14	7	*T. jamesii*	7	*T. jamesii*	0	--
*Lecanora pulicaris*	1	0	--	0	--	1	*Trebouxia* (A20)
*Lecanora stanislai*	2	0	--	1	*T. simplex*	1	*T. simplex*
*Lecanora thysanophora*	2	1	*T. jamesii*	1	*Trebouxia* (A15)	0	--
*Lecidella elaeochroma*	9	3	*T. jamesii*	3	*T. jamesii*	3	*T. jamesii, T. decolorans*
*Loxospora elatina*	2	1	*T. jamesii*	1	*Asterochloris phycobiontica*	0	--
*Melanelixia glabratula*	20	5	*T. jamesii*	6	*T. jamesii*	9	*T. jamesii*
*Menegazzia terebrata*	1	1	*Trebouxia* (A13)	0	--	0	--
*Ochrolechia bahusiensis*	3	0	--	2	*T. suecica, T. simplex*	1	*Trebouxia* (A22)
*Parmelia saxatilis*	3	1	*T. suecica*	0	--	2	*T. suecica*
*Parmelia sulcata*	6	2	*Trebouxia* (A11)	2	*Trebouxia* (A11)	2	*Trebouxia* (A11)
*Parmelia sulcata* s.l.	2	1	*Trebouxia* (A11)	0	--	1	*Trebouxia* (A11)
*Pertusaria amara*	19	8	*T. jamesii*	7	*T. jamesii*	4	*T. jamesii*
*Pertusaria coccodes*	9	5	*T. jamesii*	3	*T. jamesii*	1	*T. jamesii*
*Pertusaria leioplaca*	9	3	*T. jamesii*	5	*T. jamesii*	1	*T. jamesii*
*Pertusaria* sp. 1	4	3	*T. jamesii, Trebouxia* (A14)	1	*Trebouxia* (A14)	0	--
*Phlyctis argena*	9	1	*T. jamesii*	4	*Dictyochloropsis* sp.	4	*T. jamesii, Dictyochloropsis sp.*
*Physcia adscendens*	1	0	--	0	--	1	*T. flava*
*Platismatia glauca*	5	1	*T. suecica*	1	*Trebouxia* (A17)	3	*Asterochloris phycobiontica, T. suecica*
*Pseudevernia furfuracea*	2	0	--	0	--	2	*T. suecica*
*Pyrenula nitida*	4	4	*Trentepohlia* (A2)	0	--	0	--
*Pyrenula nitidella*	1	1	*Trentepohlia* (A2)	0	--	0	--
*Ramalina farinacea*	7	2	*T. jamesii*	1	*T. jamesii*	4	*T. jamesii*
*Ramalina pollinaria*	3	3	*T. jamesii*	0	--	0	--
*Rinodina* sp.	1	0	--	1	*Trebouxia* (A11)	0	--
*Ropalospora viridis*	1	1	*Trebouxia* (A19)	0	--	0	--
*Thelotrema lepadinum*	5	5	*Trentepohlia* (A3), *Trentepohlia* (A5)	0	--	0	--

**Table 3:** Algal OTUs: Summary of the sampled photobiont sequences, including their frequency and fungal hosts.

OTUs	Photobiont sp.	Overall Occurrence	Associated # Fungal OTU(s)	Fungal Hosts (This Study)	Clade/OTU sensu Leavitt et al. [19]	Top NCBI Hits/Other Studies
A0	*Asterochloris phycobiontica* strain SAG 26.81	11	7	*Cladonia gracilis*, *C. coniocraea*, *C*. *digitata*, *C. chlorophaea*, *C. fimbriata*, *Loxospora elatina*, *Platismatia glauca*	NA	*Cladonia symphycarpa*, *Lepraria caesioalba*, *Varicellaria carneonivea*
A1	*Trentepohlia* sp SAG11880	3	1	*Graphis pulverulenta*, *Graphis scripta*	NA	*Graphis scripta*
A2	*Trentepohlia* (A2)	4	1	*Pyrenula nitida*, *Pyrenula nitidella*	NA	None
A3	*Trentepohlia* (A3)	4	1	*Thelotrema lepadinum*	NA	*Trentepohlia* annulata strain SAG 20.94 (96.5%)
A4	*Trentepohlia* (A4)	4	2	*Graphis betulina*, *Graphis* sp. 2	NA	None
A5	*Trentepohlia* (A5)	1	1	*Thelotrema* sp.	NA	None
A6	*Trebouxia jamesii*	123	16	*Biatora* sp. *2*, *Evernia prunastri*, *Lecanora argentata*, *L. chlarotera*, *L. glabrata*, *L. thysanophora*, *Lecidella elaeochroma*, *Loxospora elatina*, *Melanelixia glabratula*, *Pertusaria amara*, *P. coccodes*, *P. leioplaca*, *P*. sp. 1, *Phlyctis argena*, *Ramalina farinacea*, *R. pollinaria*	Clade A, OTU A03	*Lecanora bicincta*, *L*. *rupicola*, *Protoparmelia badia*, *P. montagnei*, *Tephromela atra*
A7	*Parachloroidium* sp.	5	1	*Fellhanera* sp.	NA	None
A8	*Trebouxia decolorans* UTEXB781	9	2	*Lecidella elaeochroma*, *Lecanora carpinea*	Clade A, OTU A33	*Xanthoria candelaria, X. elegans*, *X. parietina*
A9	*Trebouxia suecica*/ T. *australis*	19	7	*Buellia griseovirens*, *Ochrolechia bahusiensis*, *Hypocenomyce scalaris*, *Hypogymnia physodes, Parmelia saxatilis*, *Platismatia glauca*, *Pseudevernia furfuracea*	Clade S, OTU S05	*Cetraria islandica*
A10	*Trebouxia simplex*	12	3	*Buellia griseovirens*, *Lecanora stanislai*, *Ochrolechia bahusiensis*	Clade S, OTU S10	*Bryoria furcellata*, *Boreoplaca ultrafrigida*, *Imshaugia aleurites*, *Psora decipiens*, *Protoparmelia ochrococca*, *Thamnolia vermicularis*, *Tuckermannopsis americana*, *Umbilicaria esculenta*, *Pseudevernia consocians*
A11	*Trebouxia* (A11)	9	2	*Parmelia sulcata*, *Rinodina* sp.	Clade I, OTU 102	*Melanelixia subaurifera*
A12	*Dictyochloropsis* sp.	7	1	*Phlyctis argena*	NA	None
A13	*Trebouxia* (A13)	3	2	*Cetrelia olivetorum*, *Menegazzia terebrata*	Clade A, NONE	None
A14	*Trebouxia* (A14)	2	1	*Pertusaria* sp. 1	Clade A, NONE	None
A15	*Trebouxia* (A15)	1	1	*Lecanora thysanophora*	Clade S, OTU S08	None
A16	*Trebouxia flava* UTEX181	1	1	*Physcia adscendens*	Clade I, NONE	*Physconia grisea*, *P. distorta*, *P. enteroxantha*, *Tephromela atra*
A17	*Trebouxia* (A17)	1	1	*Platismatia glauca*	Clade I, OTU I03	None
A18	*Trebouxia* (A18)	1	1	*Hypotrachyna britannica*	Clade S, NONE	None
A19	*Trebouxia* (A19)	1	1	*Ropalospora* sp.	Clade A, NONE	None
A20	*Trebouxia* (A20)	1	1	*Lecanora pulicaris*	Clade S, NONE	None
A21	*Apatococcus*	2	1	*Biatora* sp. 1	NA	None
A22	*Trebouxia* (A22)	2	2	*Buellia griseovirens*, *Ochrolechia androgyna*	Clade S/NONE	None

## Supplementary Materials

Supplementary materials can be found at https://www.mdpi.com/2076-2607/7/9/335/s1.
